# Second trimester cytokine profiles associated with gestational diabetes and hypertensive disorders of pregnancy

**DOI:** 10.1371/journal.pone.0279072

**Published:** 2022-12-14

**Authors:** Paulina M. B. Hart, Nikki L. Stephenson, Natalie V. Scime, Suzanne C. Tough, Donna M. Slater, Kathleen H. Chaput

**Affiliations:** 1 Department of Paediatrics, Cumming School of Medicine at University of Calgary, Calgary, AB, Canada; 2 Department of Community Health Sciences, Cumming School of Medicine at University of Calgary, Calgary, AB, Canada; 3 Department of Physiology and Pharmacology, Cumming School of Medicine at University of Calgary, Calgary, AB, Canada; 4 Department of Obstetrics and Gynaecology, Cumming School of Medicine at University of Calgary, Calgary, AB, Canada; University of Cambridge, UNITED KINGDOM

## Abstract

Healthy pregnancy requires a coordinated immune response, yet complications can arise, putting both the mother’s and child’s health at risk. Hypertensive disorders of pregnancy (HDP) and gestational diabetes mellitus (GDM) are pregnancy-related complications that account for most maternal morbidity and mortality. Cytokines are proteins released as part of the immune response to disease or infection and regulate inflammation. Certain pregnancy complications cause localized and systemic inflammation; however, cytokine profiles specific to such complications are not well understood. This study aims to examine associations between pregnancy complications of HDP and GDM and cytokine profiles in the second trimester of pregnancy. Data was obtained from the All Our Families birth cohort in Calgary, Alberta, Canada. The cohort collected questionnaires at the time of participant enrollment and maternal blood samples at 17–23 weeks gestation. Cases of HDP (n = 27) and GDM (n = 31) were matched to controls on BMI, maternal age, and smoking status in the preconception period at a 1:3 ratio. Cytokine levels were measured in blood samples using Luminex xMAP technology using a panel of 42 cytokines. Using R software, a Classification and Regression Tree (CART) analysis was conducted to identify cytokine profiles and levels associated with each complication. Four cytokines were identified in the HDP CART (in descending order of importance): Monocyte Chemoattractant Protein-1 (cut-off: <480pg/mL), Macrophage Inflammatory Protein-1β (cut-off: ≥26pg/mL), Eotaxin (cut-off: <27/≥27&<36/≥36pg/mL), and Soluble Cluster of Differentiation 40 Ligand (cut-off: <1342pg/mL). Six cytokine levels were identified in the GDM CART: Interleukin-1 Receptor Antagonist (IL-1Ra; cut-off: <25pg/mL), Interleukin-5 (cut-off: ≥0.4pg/mL), Interferon-γ (cut-off: <4.9pg/mL), IL-1Ra (cut-off: ≥111pg/mL), Eotaxin (cut-off: ≥21pg/mL), and Interleukin-18 (cut-off: ≥155pg/mL). By examining the complex inter-relationships between cytokines, findings of cytokine profiles guide further research in identifying biomarkers of pregnancy complications relevant to the design of the future management or prevention of these conditions.

## Introduction

The World Health Organization estimated that 810 women worldwide died every day from pregnancy and childbirth complications in 2017, many of which are preventable [1]. Hypertensive disorders of pregnancy (HDP) and gestational diabetes mellitus (GDM) account for the majority of maternal morbidity and mortality and are associated with alterations in the mother’s immune responses during pregnancy [2].

HDP are common disorders to develop during pregnancy and affect around 7% of pregnancies in Canada [3]. HDP encompasses chronic (pre-existing) hypertension, gestational hypertension, and pre-eclampsia. Gestational hypertension is diagnosed when there is new onset of systolic blood pressure of at least 140mmHg and/or diastolic blood pressure of at least 90mmHg, on two or more occasions during pregnancy manifesting after 20 weeks gestation. Many women with gestational hypertension will be diagnosed with pre-eclampsia, a multisystemic disorder diagnosed by the appearance of both hypertension and proteinuria, affecting 5–7% of pregnancies [4]. Pre-eclampsia is of particular concern as it can rapidly progress to cause health complications and can lead to both maternal and perinatal morbidity [4]. GDM is an inflammatory condition associated with carbohydrate intolerance and insulin insensitivity that is first recognized during pregnancy, affecting up to 22% of all pregnant women [5]. GDM is diagnosed when there is a one-hour glucose value greater than or equal to 11.1mmol/L. Although most women with GDM return to normal glucose levels after delivery, their child is at high risk for developing obesity, impaired glucose tolerance, and type 2 diabetes in adulthood [6].

Proteins involved in inflammation trigger signalling pathways and mediate the coordinated communications between the mother and fetus to promote a healthy pregnancy [2, 7]. Cytokines are one such family of proteins that are important in regulating immune responses, and are divided into two main groups: pro- and anti-inflammatory cytokines that activate responses to modulate the immune system [8, 9]. Cytokines produced by the placenta are thought to have an important role in maintaining a complex communication network within the feto-maternal interface to aid with growth and development [9, 10]. Previous studies have found several factors that are associated with altered cytokine levels during pregnancy, including stress, gestational age, ethnicity, smoking, and body mass index (BMI) [5].

While there are differences in clinical details of HDP and GDM, both have been linked with changes in inflammatory profiles. Previous studies suggest that elevations in inflammatory cytokines like tumour necrosis factor-alpha (TNF-α) and interleukin (IL)-1 may contribute to the development of hypertension and pre-eclampsia [8]. Studies have found associations between GDM and high serum concentrations of pro-inflammatory cytokines IL-6, IL-7, and IL-18 and lower levels of anti-inflammatory mediators IL-4 and IL-10 but other studies do not confirm this [5]. The pathogenesis is unclear, but it is believed that cytokines produced during inflammation in GDM, may cause beta cell destruction, resulting in defective insulin action [5, 6, 10].

Prevention and treatment of pregnancy complications requires knowledge of their etiology and pathogenesis as well as methods for identifying those at high risk for developing a complication [8]. Dysregulation of inflammatory responses, either exaggerated or inadequate, have been linked to perinatal diseases, including gestational hypertension, pre-eclampsia, and gestational diabetes [8]. However, previous studies have generally examined levels of individual cytokines associated with these conditions, rather than considering the complex inter-relationships between cytokines that may be involved in the disruption of a healthy immune balance during pregnancy. Although pregnancy involves complex changes in physiology throughout all gestational periods, by investigating potential predictors early on in pregnancy, such knowledge may help to identify potentially modifiable biomarkers relevant to the design of future management or prevention of these conditions.

Therefore, the objective of the current study was to examine associations between pregnancy complications of HDP and GDM and cytokine profiles in the second trimester of pregnancy. Through exploratory methods in our study, the aim of our investigation was achieved.

## Materials and methods

### Data source

Data were obtained from the All Our Families (AOF) Study, a longitudinal community-based pregnancy cohort in Calgary, Alberta, Canada, designed to investigate the relationship between prenatal and early-life periods and outcomes for infants, children, and families. The cohort recruited 3200 pregnant women in Alberta between 2008–2010. Eligible women for the cohort study were less than 25 weeks of gestation at enrollment, at least 18 years of age, accessing prenatal care in Calgary, and able to understand written and spoken English [11]. A questionnaire at the time of enrollment was administered to collect information on socio-demographic characteristics, pregnancy history, psychosocial health, as well as delivery and birth outcomes [12]. A subgroup within the cohort (n = 1871) provided blood samples at 17–23 and 27–33 weeks gestation [12]. Whole blood was collected by trained phlebotomists in 10mL sterile glass tubes with no additives, and immediately centrifuged to separate plasma, which was then frozen in a dedicated 30°C freezer. Participants provided written informed consent for the original cohort study. This secondary analysis was approved by the Conjoint Health Research Ethics Board at the University of Calgary (REB18-2017), and participants were not required to provide additional consent for this secondary analysis. The ethics agreement and participant consent from the original cohort study prohibits study data from being publicly available; however, study data are made available from the University of Calgary (allourfamilies@ucalgary.ca) subsequent to proposal submission and ethics review for researchers who meet the specific criteria for access to confidential data.

### Case-control matching

For this analysis, blood plasma samples collected at 17–23 weeks gestation were studied to examine the earliest available measure of cytokines associated with the presence of pregnancy complications. Pregnancy complication status was obtained by accessing electronic medical records with the mother’s consent from the Alberta Perinatal Health Program database, a province-wide database with prenatal maternal and birth outcome information for all births in Alberta [11]. Diagnostic ICD10-CA coding was used to identify cases of GDM (code O24.8) and HDP which included gestational hypertension (code O13) and pre-eclampsia (code O14). Chronic hypertension cases were included in the HDP group and identified by the discharge abstract from the labour and delivery records. Controls were women without diagnostic codes nor record for these pregnancy complications, matched 3:1 to cases on pre-pregnancy smoking status (yes, no), pre-pregnancy BMI category of: underweight (<18.5 kg/m^2^), normal weight (18.5–24.9 kg/m^2^), overweight (25.0–29.9 kg/m^2^), and obese (≥30.0 kg/m^2^), and maternal age group at delivery (<25 years, 25–34 years, ≥35 years). These matching variables were chosen to control for confounding based on previous research on pregnancy complications and cytokine profiles [13, 14]. Each control was matched to only one case. There were 31 cases of GDM matched to 93 controls and 27 cases of HDP matched to 81 controls.

### Cytokine measurement

Maternal blood plasma samples collected between 17–23 weeks gestation were aliquoted and used to assess cytokine levels. We used Luminex xMAP technology for multiplexed quantification of 42 human cytokines, chemokines, and growth factors. The multiplexing analysis was performed using the Luminex^TM^ 200 system (Luminex, Austin TX, USA) by Eve Technologies Corporation (Calgary, Alberta). In the parent study from which we extracted our data, duplicate assays were performed on 700 samples in 14 96-well microplates using Eve Technologies’ Human Cytokine 42-Plex Discovery Assay® (catalogue #HCYTOMAG-60K, MilliporeSigma, Burlington, Massachusetts, USA), according to the manufacturer’s protocol. A 70-sample (10%) pilot assay was run to calibrate settings and ensure that cytokine signals fell in the dynamic range of the standard curve, following which, no plate-to-plate adjustments were made. Assay sensitivity values (minimum detectable concentrations) are available in the MilliporeSigma MILLIPLEX® MAP protocol (S1 Table) [15]. The mean concentrations of cytokine levels from the two replicate assays were used for statistical analysis [16]. The following 42 cytokines, chemokines, and growth factors were simultaneously measured: epidermal growth factor (EGF), fibroblast growth factor (FGF), eotaxin (CCL11), transforming growth factor-alpha (TGF-α), granulocyte colony stimulating factor (G-CSF), Fms related tyrosine kinase 3 ligand (Flt-3L), granulocyte-macrophage colony stimulating factor (GM-CSF), fractalkine, interferon (IFN)-α, IFN-γ, growth related gene product (GRO) (CXCL1), IL-1α, IL-1ß, IL-2, IL-3, IL-4, IL-5, IL-6, IL-7, IL-8 (CXCL8), IL-9, IL-10, IL-12, IL-12P70, IL-13, IL-15, IL-17A, IL-18, interleukin-1 receptor antagonist (IL-1Ra), monocyte chemotactic protein (MCP)-3 (CCL7), MCP-1 (CCL2), macrophage-derived chemokine (MDC) (CCL22), soluble cluster of differentiation 40 (sCD40L), macrophage inflammatory protein (MIP)-1α (CCL3), MIP-1ß (CCL4), TNF-α, TNF-ß, interferon gamma-induced protein 10 (IP-10) (CXCL10), vascular endothelial growth factor (VEGF)-α, platelet-derived growth factors (PDGF-AA, PDGF-AB/BB), and RANTES. We excluded data for PDGF-AA, PDGF-AB/BB, and Rantes, as these could not reliably be measured in our undiluted samples.

### Statistical analysis

Analyses for each pregnancy complication were carried out separately using R version 4.1.0 with RStudio1.4.1717. First, we used descriptive statistics to describe sample characteristics (i.e., matched variables) and cytokine distributions according to case and control groups. The “mytable” function with “ztable” output for formatting was computed for data exploration to determine the median and interquartile range of each cytokine level in all groups. Next, we conducted a classification and regression tree (CART) analysis. CART was used to determine which of the 39 cytokines included, and which combinations, were associated with each of the outcomes and at which cut-off values. CART is a non-parametric statistical method that uses a data-driven approach to separate a set of observations into subgroups that are increasingly homogenous with respect to the outcome of interest, and which captures inter-relationships between exposures [16]. CART allows for the analysis of relationships between large numbers of variables, with no underlying knowledge or assignment of exposure groups, such as normal or abnormal biological ranges, which are currently unknown in pregnancy, precluding meaningful comparisons using traditional categorical statistical comparisons. Unlike traditional regression methods, CART does not require underlying assumptions about data distributions or variable relationships (i.e., a normal distribution or linear association), making it ideal for this analysis, given that biomarker levels are generally not normally distributed, and linearity of relationships between numerous variables is difficult to determine. The tree starts with a parent node (“root”) that contains all observations in the sample; then, the algorithm searches for the best binary split based on similar outcomes to create child nodes (“leaves”) [16]. The estimated proportion of individuals with the outcome in the child nodes reflects the probability of having the dependent measure in the sample [16]. As the tree continues to split, child nodes will each become the parent to two more child nodes. When no further splits are made, terminal nodes are created. We used the “rpart” package with standard defaults for determining tree size and splitting based on optimization of the Gini index to conduct our CART analyses [17].

## Results and discussion

### Samples and cytokines excluded

Of the 42 cytokines that were identified to be present within the maternal plasma at 17–23 gestational weeks, 39 were appropriately measured and included for analysis. Three cytokines, including two variants of platelet-derived growth factor (PDGF-α and PDGF-β) and the RANTES chemokine (regulated on activation, normal T-cell expressed and secreted) were excluded from the analysis as they could not reliably be measured without dilution and thus could not be measured in our samples. Additionally, the samples of five participants were excluded from analysis, prior to control selection, for suspected human anti-animal antibody (HAAA) exposure, due to unexpectedly high results of cytokine levels. HAAAs are human antibodies that are produced when a person is exposed to an animal monoclonal antibody, and can produce artificially high cytokine signals in immunoassays, creating extreme-outlier readings.

### Participants

In total, 232 mothers were included. The summary of participant characteristics in the HDP case and control set is provided in Table 1. There were 27 participants in the HDP case group (2 pre-eclampsia, 23 gestational hypertension, and 1 chronic hypertension) with 81 matched controls. The majority of the participants in this case-control set were overweight (33.3%) or obese (33.3%), non-smokers (88.9%), and between 18–35 years old at delivery (81.5%).

**Table 1:** Summary of characteristics that matched those with HDP or GDM to their respective control groups.

Participant Characteristics	HDP Controls	HDP Cases	GDM Controls	GDM Cases
	(N = 81)	(N = 27)	(N = 93)	(N = 31)
	N (%)	N (%)	N (%)	N (%)
Pre-pregnancy BMI category				
Underweight	3 (3.7)	1 (3.7)	0 (0.0)	0 (0.0)
Normal	24 (29.6)	8 (29.6)	33 (35.5)	11 (35.5)
Overweight	27 (33.3)	9 (33.3)	21 (22.6)	7 (22.6)
Obese	27 (33.3)	9 (33.3)	39 (41.9)	13 (41.9)
Pre-pregnancy smoking status				
Yes	9 (11.1)	3 (11.1)	12 (12.9)	4 (12.9)
No	72 (88.9)	24 (88.9)	81 (87.1)	27 (87.1)
Maternal age at delivery				
<18	3 (3.7)	1 (3.7)	0 (0.0)	0 (0.0)
18–35	66 (81.5)	22 (81.5)	54 (58.1)	18 (58.1)
35 and over	12 (14.8)	4 (14.8)	39 (41.9)	13 (41.9)

The summary of participant characteristics in the GDM case and control set is provided in Table 1. There were 31 participants in the GDM case group with 93 matched controls. The majority of participants in this case-control were obese (41.9%), non-smokers (87.1%), and between 18–35 years old (58.1%) or over 35 years old (49.1%) at delivery.

### CART results

**Fig 1:**
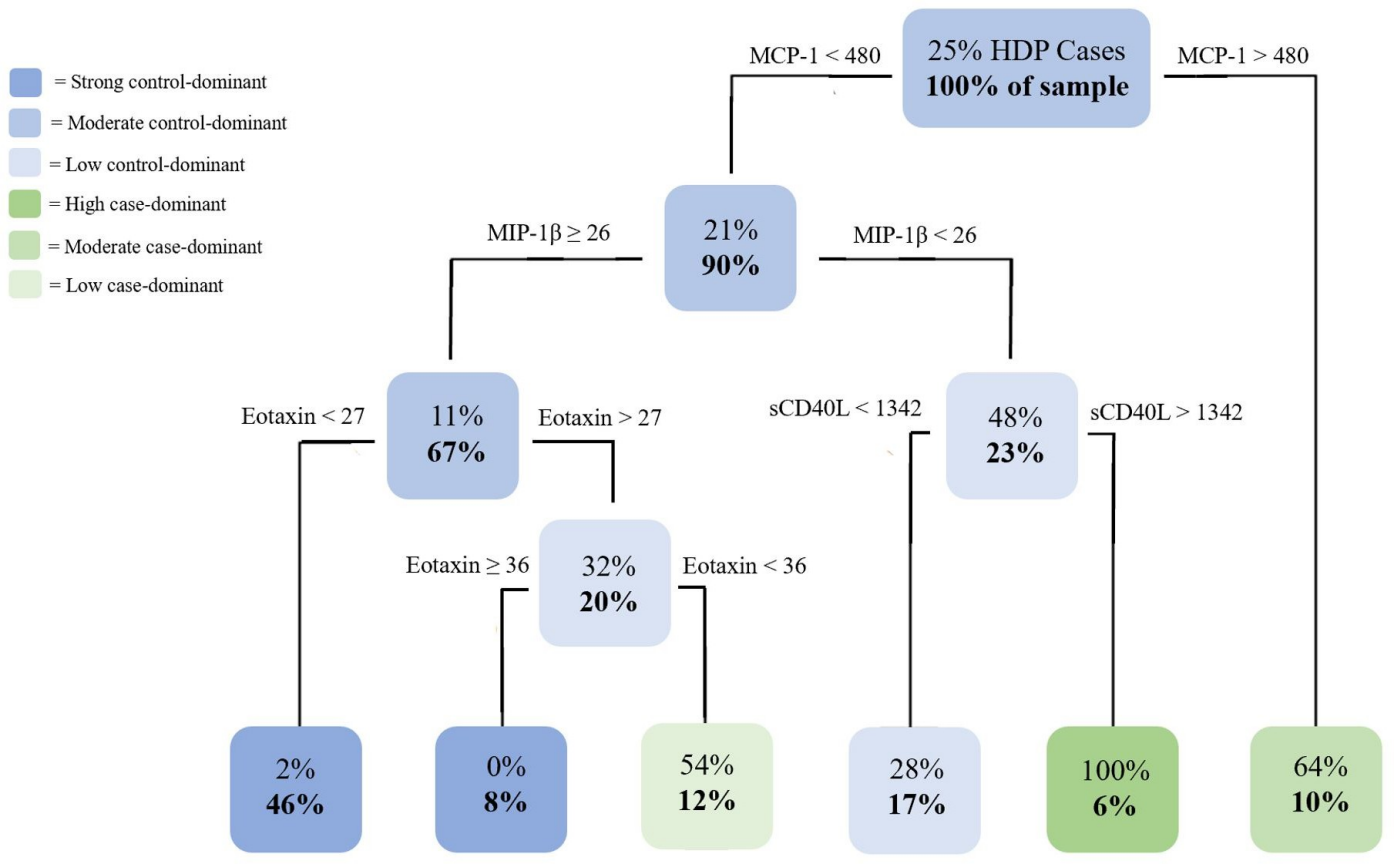
CART analysis of cytokine levels of participants within the HDP subset. Analysis of the HDP subset (n = 108) representing the probability of disorder diagnosis and percentage of the sample size using R software. Specific cytokines and cut-off levels (pg/mL) that are the most important in distinguishing cases versus controls were identified with colours and shading representing the likelihood of condition diagnosis.

**Fig 2:**
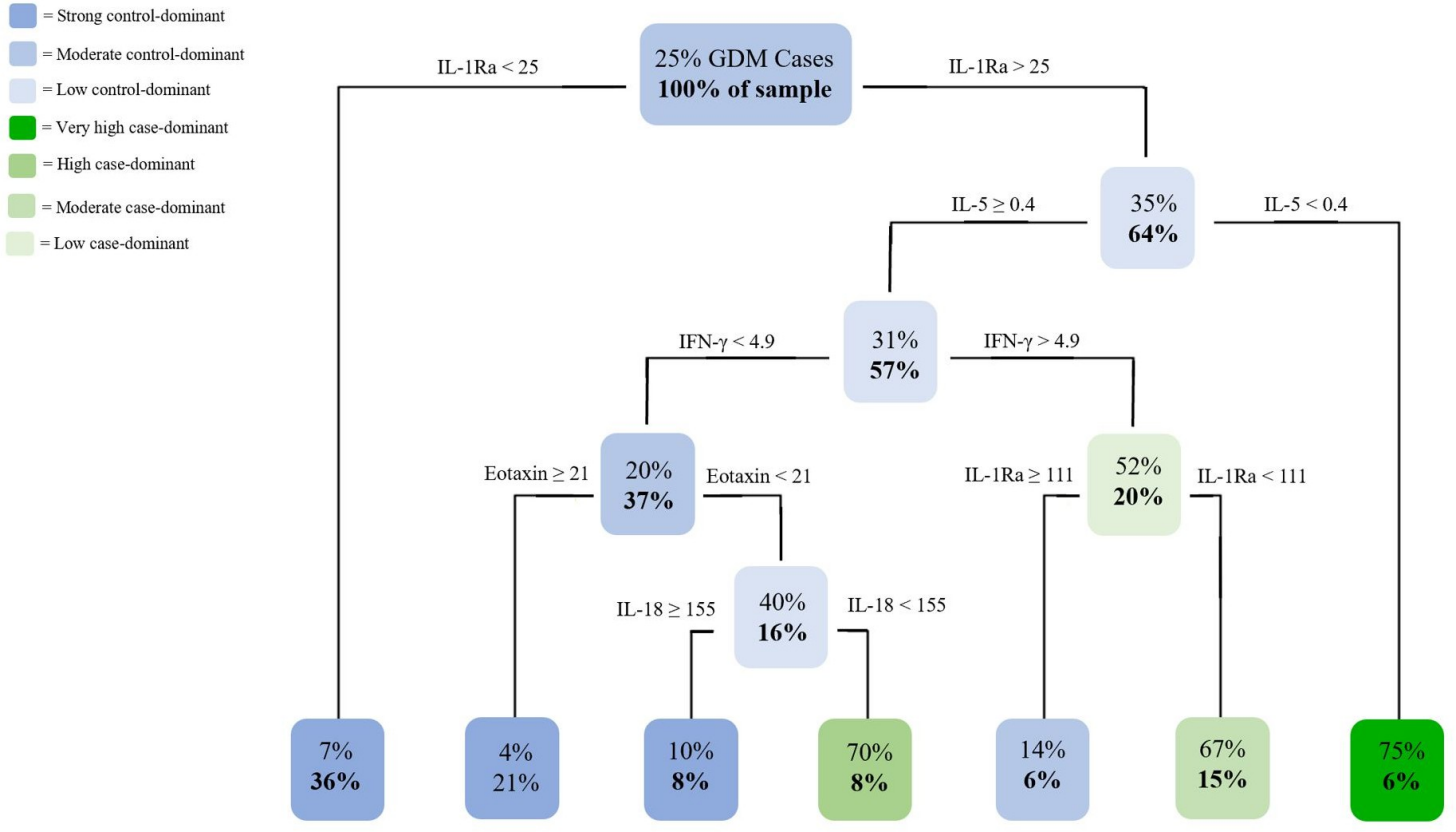
CART analysis of cytokine levels of participants within the GDM subset. Analysis of the GDM subset (n = 124) representing the probability of disorder diagnosis and percentage of the sample size using R software. Specific cytokines and cut-off levels (pg/mL) that are the most important in distinguishing cases versus controls were identified with colours and shading representing the likelihood of condition diagnosis.

The mean and standard deviation of each cytokine in cases versus controls was computed for data exploration in each pregnancy complication set (Table 2). Separate CART analyses were conducted for each set (Figs 1 and 2).

**Table 2:** Summary of the median and interquartile range (IQR) of cytokine levels (pg/mL) in plasma of those with HDP or GDM and those without complication.

Cytokine	HDP Controls	HDP Cases	GDM Controls	GDM Cases
	(N = 81)	(N = 27)	(N = 93)	(N = 31)
	Median (IQR)	Median (IQR)	Median (IQR)	Median (IQR)
EGF	28.1 (65.8)	39.0 (64.8)	25.0 (69.6)	48.0 (86.4)
Eotaxin	22.1 (8.8)	27.4 (13.8)	22.4 (10.0)	21.9 (16.9)
FGF	68.8 (31.0)	70.6 (36.4)	80.0 (33.5)	81.5 (28.6)
Flt-3L	4.0 (17.5)	4.2 (24.2)	5.0 (21.1)	5.2 (17.2)
Fractalkine	38.5 (64.3)	69.1 (76.0)	51.6 (60.8)	41.4 (70.2)
G-CSF	26.5 (14.5)	25.7 (42.5)	24.6 (21.8)	30.0 (40.5)
GM-CSF	10.7 (12.4)	15.1 (11.9)	12.0 (12.3)	16.0 (14.9)
GRO	698.6 (540.8)	758.2 (891.0)	854.5 (674.4)	1,001.5 (895.0)
IFN-α	19.9 (9.5)	21.3 (19.1)	22.7 (11.0)	24.2 (20.9)
IFN-γ	3.2 (4.1)	3.0 (2.7)	3.3 (4.0)	4.4 (3.9)
IL-10	1.6 (2.0)	1.7 (1.6)	1.5 (2.2)	1.6 (2.5)
IL-12	7.3 (13.9)	13.7 (19.0)	8.2 (16.7)	10.4 (27.5)
IL-12P70	3.4 (2.8)	3.3 (1.2)	3.4 (2.4)	3.7 (4.7)
IL-13	1.3 (3.9)	2.0 (3.5)	1.5 (3.7)	1.2 (7.9)
IL-15	3.5 (2.4)	4.3 (2.0)	3.9 (2.1)	4.9 (5.1)
IL-17A	3.4 (3.6)	2.6 (4.6)	3.4 (3.7)	3.0 (3.0)
IL-18	143.9 (322.1)	246.5 (390.7)	114.9 (368.3)	143.9 (405.9)
IL-1a	16.6 (62.0)	32.9 (80.3)	16.9 (61.9)	20.3 (119.7)
IL-1B	0.9 (0.5)	1.0 (1.4)	1.0 (1.3)	1.3 (2.8)
IL-1RA	28.5 (33.6)	43.7 (55.2)	27.8 (65.8)	54.0 (57.7)
IL-2	0.6 (0.4)	0.7 (0.3)	0.7 (0.6)	0.8 (1.1)
IL-3	0.4 (0.0)	0.4 (0.1)	0.4 (0.0)	0.4 (0.1)
IL-4	8.3 (20.4)	17.5 (34.0)	8.3 (24.4)	15.1 (39.9)
IL-5	0.5 (0.1)	0.5 (0.1)	0.5 (0.1)	0.5 (0.3)
IL-6	2.0 (4.5)	2.1 (6.5)	1.5 (4.9)	3.2 (6.6)
IL-7	2.1 (2.4)	2.7 (2.5)	2.3 (2.6)	3.0 (2.3)
IL-8	4.8 (6.1)	5.3 (7.4)	5.4 (9.7)	5.3 (14.3)
IL-9	1.0 (3.1)	1.9 (4.3)	1.0 (3.5)	2.1 (4.6)
IP-10	85.6 (29.6)	84.5 (15.2)	85.4 (25.0)	88.9 (18.9)
MCP-1	220.3 (237.7)	325.1 (293.2)	230.2 (191.3)	221.9 (198.6)
MCP-3	17.4 (24.1)	19.1 (35.8)	18.2 (30.1)	27.4 (44.3)
MDC	483.9 (141.2)	507.3 (218.4)	475.1 (165.1)	505.7 (376.2)
MIP-1a	3.7 (1.8)	3.6 (1.8)	3.8 (1.6)	4.6 (3.8)
MIP-1B	42.0 (51.2)	47.8 (52.7)	41.7 (39.0)	42.9 (45.8)
sDC40L	874.5 (1,598.7)	1,865.4 (1,715.6)	928.1 (1,674.6)	940.2 (1,596.5)
TGF-α	3.3 (3.8)	3.2 (3.5)	3.4 (4.5)	3.8 (6.5)
TNF-a	13.1 (10.4)	17.0 (6.1)	15.1 (8.2)	16.4 (10.2)
TNF-B	2.8 (39.5)	19.3 (70.3)	3.7 (33.1)	12.0 (80.8)
VEGF-a	9.6 (11.4)	7.2 (11.8)	12.0 (11.4)	15.8 (12.9)

CART trees include root (top), branch (middle), and terminal (bottom) nodes. Terminal nodes indicate the probability of classification within the sample, if all conditions (i.e. cut-points) are met in each of the nodes above it. The HDP tree (Fig 1) produced three unique cytokine profiles with six terminal nodes including 3 case-dominant (green nodes) and 3 control-dominant nodes (blue nodes) to classify a woman’s HDP status. The profiles were based on the following cytokines: MCP-1, MIP-1β, Eotaxin, and sCD40L. Women in our sample had a 100% probability of HDP diagnosis if they had MCP-1 levels <480pg/mL, MIP-1β levels <26pg/mL, and sCD40L levels >1342pg/mL (6% of sample). Additionally, women were more likely to have HDP if they had MCP-1 levels >480pg/mL (64% HDP cases; 10% of sample) or if they had MCP-1 levels <480pg/mL, MIP-1β levels ≥26pg/mL, and intermediate Eotaxin levels between 27-36pg/mL (54% HDP cases; 13% of sample).

The GDM tree (Fig 2) produced three unique cytokine profiles with 7 terminal nodes including 3 case-dominant (green nodes) and 4 control-dominant nodes (blue nodes) to classify a woman’s GDM status. The 7 profiles were based on the following cytokines: IL-1Ra, IL-5, IFN-γ, IL-18, and Eotaxin. Women in our sample had a 75% likelihood of GDM diagnosis if they had IL-1Ra level >25pg/mL and IL-5 level <0.4pg/mL (6% of sample). Women were likely to have GDM if they had IL-1Ra level >25pg/mL, IL-5 ≥0.4pg/mL, IFN-γ level < 4.9pg/mL, Eotaxin level <21pg/mL, and IL-18 level <155pg/mL (70% GDM; 8% of sample). Women with IL-1Ra level >25pg/mL, IL-5 ≥0.4pg/mL, IFN-γ level >4.9pg/mL, and IL-1Ra level <111pg/mL had 67% probability of GDM diagnosis (15% of sample).

### Principal findings

This exploratory study revealed insight into the inter-relationships between cytokine levels and found a profile of 4 and 6 cytokine clusters distinct to both HDP and GDM respectively, providing a novel approach for identifying inflammatory response profiles in two pregnancy disorders warranting further research.

When comparing cases of HDP to controls, the relationship between levels of MCP-1, MIP-1β, Eotaxin, and sCD40L were identified as critical cytokines involved in predicting condition diagnosis in our sample (Fig 1). MCP-1 is a potent chemoattractant for monocytes and macrophages to areas of inflammation, being observed in tissues during inflammation-dependent disease progression [18]. A previous study found 1.5-fold higher levels of MCP-1 in women with pre-eclampsia compared with healthy pregnant women, suggesting that MCP-1 can serve as a potential clinical marker for pre-eclampsia [19]. This was observed in our results, as those with higher MCP-1 levels at 17–23 weeks gestation had a moderate probability of HDP diagnosis later in pregnancy. However, those with low MCP-1 but with specific levels of MIP-1β, Eotaxin, and sCD40L had a higher probability of HDP diagnosis, which further substantiates the role of MCP-1 in pre-eclampsia pathology.

Fig 1 shows that while also considering MCP-1 and MIP-1β levels, individuals with moderate Eotaxin levels (27-36pg/mL) had the greatest likelihood of HDP diagnosis in comparison to the profiles of women with high (≥36pg/mL) or low (<27pg/mL) levels. Eotaxin is a peptide immune modulator that regulates the chemoattraction of leukocytes [20]. A previous study in mice discovered a role for Eotaxin in the pathogenesis of many inflammatory conditions, suggesting a potential role in modulating human inflammation and health complications [21].

The GDM tree in Fig 2 identified IL-1Ra, in addition to IL-5, IFN-γ, Eotaxin, and IL-18, as the most important cytokines involved in predicting GDM diagnosis in our sample. IL-1Ra is an anti-inflammatory cytokine that acts as an antagonist to the pro-inflammatory cytokine IL-1 [22]. Previous research suggests an association between diabetes incidence and dysregulation of IL-1 and IL-1Ra [22]. Studies have found that GDM patients have significantly lower levels of IL-1Ra when compared to controls [23]. We observed that low levels of IL-1Ra (<25pg/mL) were associated with a low likelihood of GDM diagnosis, which is inconsistent with previous research. However, when looking at the other side of the split when considering IL-1Ra in relation to IL-5 and IFN-γ, splitting based on IL-1Ra levels occurs again to produce different likelihoods of GDM diagnosis based on a range of IL-1Ra levels.

Previous research and knowledge on individual cytokines provide insight on the profile of women at risk of HDP or GDM; however, the ability to predict pregnancy complications may be improved by understanding the intersection of various cytokine values. Because this is an exploratory study, additional research is needed to confirm the findings, but our study provides insight into the importance of cytokine profiles and its potential in clinical practice.

While this is a topic for further research in the context of human pregnancy complications, taken together, our findings with Eotaxin and IL-1Ra suggest the need to consider different roles cytokines may have when in different ranges of levels (low, moderate, and high) in relation to others. Results from this study emphasize the importance of investigating the levels and balance of cytokines in relation to others in establishing patterns which can be missed when levels of single cytokines are studied. Although our analysis did find cytokines in common with previous research, our study adds the element on how these cytokines may be interacting with each other in previous conditions whereas previous research does not identify significant levels or potential interactions between the cytokines identified in this study.

### Strengths and limitations

The administrative data used for GDM and HDP diagnosis is limited in that the timing of diagnosis is unknown. We are unable to distinguish between early versus late onset of pregnancy complications or to determine the time gap between cytokine measurement and clinical diagnosis. The blood was drawn from our participants between 17–23 weeks, which precedes the timeframe during which HDP and GDM are typically diagnosed. However, evidence suggests that physiologic changes likely occur prior to clinical diagnosis, thus the assays may be detecting subtle changes that occurred prior to diagnosis [24]. Further, these conditions can take weeks or months to diagnose, including multiple laboratory tests (GDM) and at least two consecutive high blood pressure readings (HDP). We acknowledge that there may have been samples included that are misclassified as blood was drawn prior to disease onset. Should this have occurred, our findings would be biased towards the null, and our conclusions would be conservative.

Confounding variables are important to consider. A study investigating factors that influence cytokine responses found that concentrations of IL-6 and IL-1Ra are increased in the circulation of older individuals [26]. We accounted for this by matching cases by maternal age at delivery. We also matched cases by pre-pregnancy smoking status, and maternal BMI, which are known to affect the population of cytokines [5]. Race/ethnicity appears to be associated with differential cytokine levels; however, we did not control for this variable as the majority of women were white. Further research is needed to explore racial variability to further understand the intersection between social determinants of health, pregnancy complications, and immune system responses in pregnancy. Additionally, as we could not control for all confounding variables, further studies should attempt to explore other covariates through regression analysis while at the same time being aware of overmatching when controlling for too many variables.

Blood samples included were measured between week 17–23 of gestation. Cytokine concentration changes occur throughout pregnancy, and it is possible for random variability to be introduced as measurements were taken differentially across a 6-week period [25]. Spence et al. found initial observations of TNF-α increasing throughout pregnancy and IL-8 decreasing in the second trimester [25]. Additionally, the time of day and year were not controlled for during data collection. A study by Altara et al. found that plasma collected in the afternoon contained higher concentrations of cytokines than serum and plasma collected in the morning and serum collected in the afternoon, suggesting that cytokines have a diurnal rhythm of release [26]. Additionally, it has been found that there is a significant seasonality pattern of immune responses with TNF-α, IL-1β, and IL-6 peaking in the summer [27]. Although it is difficult to isolate single associations to cytokine levels as seasonal allergies may contribute to the changes observed in the seasonality patterns of cytokines, it is important to consider the contribution of confounding on cytokine levels.

Owing to low numbers of HDP types, we homogenized pre-eclampsia and gestational hypertension into one category, as has been done previously in other studies [28]. Findings from a Norwegian cohort study found that the serum cytokine profile of women in early pregnancy who later developed pre-eclampsia was different than those who developed gestational hypertension, suggesting distinctive features of these complications [29]. As the cases of pre-eclampsia is relatively small in comparison to the majority of individuals included in the HDP group, we do not anticipate that this influenced our results in this particular analysis. However, it would be of value to repeat this type of analysis with only cases of diagnosed pre-eclampsia.

## Conclusions

This exploratory study provides insight into inflammatory profiles distinct to HDP and GDM, two common pregnancy complications. These findings revealed a cluster of specific cytokines that co-occur in each condition and are distinct from one another. Cytokine patterns may be co-dependent in the maintenance of health during pregnancy; conversely, when dysregulated, these patterns may allude to the risk of complications. Our results provide a basis for further research on cytokines and the role of inflammation in identifying those at risk for pregnancy complications.

## Supporting information

S1 Table Assay sensitivities of each cytokine. (DOCX)
