# Supplementary material for: Second trimester cytokine profiles associated with gestational diabetes and hypertensive disorders of pregnancy
Source: PLoS One. 2022 Dec 14;17(12):e0279072. doi: 10.1371/journal.pone.0279072 (PMC9749996; doi:10.1371/journal.pone.0279072)
Supplement: S1 Table — (DOCX) [file pone.0279072.s001.docx]

| Cytokine | MinDC (pg/mL) | MinDC+2SD (pg/mL) |
| --- | --- | --- |
| EGF  FGF  Eotaxin  TGF α  G-CSF  Flt-3L  GM-CSF  Fractalkine  IFN α2  IFN γ  GRO  IL-10  MCP-3  IL-12P40  MDC  IL-12P70  IL-13  IL-15  sCD40L  IL-17  IL-1RA  IL-1 α  IL-9  IL-1 β  IL-2  IL-3  IL-4  IL-5  IL-6  IL-7  IL-8  IP-10  MCP-1  MIP-1 α  MIP-1 β  TNF α  TNF β  VEGF  PDGF-AA  PDGFAB-BB  RANTES | 2.8  7.6  4.0  0.8  1.8  5.4  7.5  22.7  2.9  0.8  9.9  1.1  2.8  7.4  3.6  0.6  1.3  1.2  5.1  0.7  8.3  9.4  1.2  0.8  1.0  0.7  4.5  0.5  0.9  1.4  0.4  8.6  1.9  2.9  3.0  0.7  1.5  26.3  0.4  2.2  1.2 | 4.6  11.8  6.8  1.2  3.3  7.0  15.0  37.7  4.8  1.1  14.1  1.6  6.4  12.7  7.1  1.0  1.9  1.7  9.9  1.2  17.1  12.6  2.0  1.0  1.6  1.0  7.1  0.7  1.3  2.4  0.7  14.0  3.4  6.2  4.8  1.1  1.9  47.9  0.7  2.7  1.9 |

**S1 Table. Assay sensitivities of each cytokine**
